# Supplementary material for: Group antenatal care positively transforms the care experience: Results of an effectiveness trial in Malawi
Source: PLoS One. 2025 Jun 18;20(6):e0317171. doi: 10.1371/journal.pone.0317171 (PMC12176302; doi:10.1371/journal.pone.0317171)
Supplement: Attachment — Submitted filename: ResponseRvws_5.13.25.pdf [file pone.0317171.s002.pdf]

We thank the editor and the reviewers for their thoughtful review of our manuscript. The abstract now shows how estimates are expressed. To summarize, in the revised submission, we clarified the use of regression and logistic regression models and made the necessary distinctions between continuous and categorical outcomes. We added footnotes indicating which covariates were included to be more easily accessible to readers. We have added statistical tests for clinical outcomes and provided the p-values. We adjusted the text to distinguish between general and specific descriptions of Group ANC. We added information to address concerns about confidentiality and privacy by expanding on these topics in the methods and discussion sections. We added to the discussion about potential spillover effects, external disruptions, and limitations related to rare clinical events. For readability and conciseness, we tried to reduce repetitive statements without losing key messages. We feel that we have thoroughly addressed critiques and suggestions, including minor edits and rephrasing (e.g., language consistency). We provide detailed responses and locate major changes to each reviewer's critiques in the table below.

**Editor**	**Response**	**Section, page, or lines**
In the methods, "Statistical Analysis" section from line 248, your regression analysis approaches are not clearly described.	We clarified the regression or logistic regression approaches in the Statistical analysis section.	See Statistical Analysis, Lines 279 - 294
The last column of your regression results (from Table 3) indicate that the coefficients are either "Estimates" or odds ratios. However, for the continuous variables, even if the estimates are from the logistic regression models, the relative risks are still odds ratios.	For continuous outcome variables, regression models were employed. The estimated coefficients for Group ANC effect were in the scale of the outcome variable. For categorical outcome variables, logistic regression models were employed. The effect of Group ANC was reported as odds ratios and their 99% confidence intervals. To make the results less confusing, we condensed these results tables into 2 tables to reflect continuous (Table 3)and categorical variables (Table 4) separately. We added color coding to link the variables to the theory of change.	See Tables 3 and 4
If view of #1 above, did you use two separate regression models ("Regression/Logistic Regression"), and if so, why? Logistic regression would have been sufficient, unless your outcome was continuous. Otherwise, were the variables in the first column your outcomes? please use footnotes (see #3 below) to provide necessary descriptions for clarity.	See above and note that footnotes were added for clarity.	See Tables 3 and 4
**Abstract, Findings**		
In the "Findings" of your abstract, you reported the parameters as "Estimates". Are these ORs, or both ORs and Coefficients? ORs are also estimates. Please see how to make this clearer for the readers.	Point estimates are reported for continuous outcomes using linear regression models, and odds ratios are reported for categorical outcomes using logistic regression models. We clarified this in both the statistical analysis section and the result tables. We adjusted the abstract as well. It now includes these sentences, "Adjusted for seven individual and two clinic-level baseline covariates, point estimates are reported for continuous outcomes using multivariable linear regression models. Adjusted for the same covariates, odds ratios are reported for categorical outcomes using logistic regression models."	See Abstract, Findings
**Other**		
Indicate which covariates were adjusted for in your analyses. Consider using table footnotes to provide additional information about your regression analyses.	Footnotes were added to the new Tables 3 and 4 to indicate that regression analyses included all the individual-level and clinic-level covariates: age, parity, in a relationship, education, food security, ownership index, income quartile, client-to-midwife ratio, and community catchment area.	See Tables 3 and 4
For the clinical outcomes in Table 6, why not also report the findings as you have done for	We did statistical tests for all our planned outcomes in the proposal. The outcomes in the clinical outcomes table were no exception. Because these were relatively rare events, and the *p*-values	See Table 4

Tables 3-5? Yet, this would not apply where there are very small cases for regression analyses, as the parameters will be inestimable. Nevertheless, Reviewer 2 raises some issues with respect to these results.	were far from being significant, we did not present the p-values in the original draft. Now we have added those *p*-values and combined those results as clinical outcomes in Table 4, as suggested by Reviewer 1.	
**Reviewer 1**		
MINOR		
Abstract: why 4 OR 8 visits?	Removed from abstract because it was unclear that Ministries of Health use these measures to assess coverage and for policy purposes. Coverage is measured in multiple ways because of the 2016 WHO ANC recommendation to increase the recommended number of visits from four to eight. Governments are reporting both until 8 is routinized.	See Abstract
Line 66: remove attend the	Corrected	
Line 73: clarify that standard initial ANC visit is one-on-one/individual<br>times?	Added one-on-one to clarify	
Line 75-76: are these times inclusive of wait	Added "Excluding any wait time, Group ANC…	
Line 74-78: is group ANC conducted at the same health centre, or offsite?	Added "…conducted in the same clinics…" to clarify	
Line 80: are there non-pregnant group members in ANC? are guardians or male partners part of group ANC?	Added "Partners and non-pregnant members can also accompany the pregnant client."	
Line 140-142: move to discussion	Moved to discussion	See Discussion
Line 124: most HTC among women in Malawi probably happens through/during ANC. Is HTC not HIV prevention? Or because it happens at a different part of the health center, is it not considered ANC?	Rewritten as "Even though Malawi has a strong prevention of mother-to-child transmission program and HIV testing and counselling are a part of care, deep discussion of HIV prevention is not typically integrated into the didactic ANC health lectures."	See Lines 128-130
Throughout: Consistency... group ANC or Group ANC?	Changed all to Group ANC	
Line 240: I suggest "Malawi Kwacha" equivalent of five USD...	Changed to, "To reduce bias, participants were not compensated for ANC visits, they were offered the Malawi Kwacha equivalent of five US dollars for the time and effort it took to complete the survey."	See Procedure, Lines 266-269
Line 283: suggest allowable (or per protocol) rather than acceptable	Changed to allowable	See Line 317
Line 139: what is the sample size estimate for >99% power? It is in the protocol paper but would be important to indicate it here as well, so that readers know where the 1887 is in the ballpark or not.	The sample size that was proposed in the proposal was added in the study method section as suggested	See Methods, lines 141-144
Line 171: what was the eligibility criteria? Or do readers need to visit the protocol paper?	Added: Those under age 15, more than 24 weeks pregnant, or unable to make an informed choice (e.g., unable to converse about the study) were not ineligible."	See Participant recruitment, enrollment, and randomization

		section starting on line 170
**Major**		
Paper is well-written. It could be reviewed and shortened for readability, by removing repetition. For example, group ANC is described both in the introduction and methods. I think the authors' idea was to describe group ANC in general in the intro and then the Malawi adaptation in the methods?	Thank you. We tried to reduce redundancies. We keep both the introduction and methods descriptions of Group ANC because we describe the history and development in the introduction and then provide the specifics reflecting the model as implemented in Malawi. We do this because, in our review of the literature, it is often not clear if people are implementing the original model or if they are adapting it. This is to ensure that the reader knows exactly what the Malawi Group ANC looked like and was testing we provided these details.	See Introduction and Study Interventions sections
It would be helpful to clarify that no individual level ANC assessments were missed due to participation in group ANC.	We added this to reinforce this important point: Both Individual ANC (control) and Group ANC (intervention) adhered to the Ministry of Health guidelines. Regardless of random assignment, the health assessments and services provided to clients were expected to follow these guidelines. The two study arms are described in detail below.	See Study Interventions section
It would be good to state explicitly (if true) that women were not compensated for ANC visits (individual or group) unless it was a visit during which ACASI survey was planned. This would address a potential source of bias.	We addressed this by rewriting the sentence to say, "To reduce bias, participants were not compensated for ANC visits, they were offered the Malawi Kwacha equivalent of five US dollars for the time and effort it took to complete the survey."	See Procedure, starting at line 262
The 5260 should please be broken down by refused versus ineligible (it's in Fig 1, but please add to text). More information on both should be provided IF available. Why ineligible? And did anyone who refused/declined provide a reason? For example, if someone said I don't have time for group ANC or a friend was in group ANC before and didn't like it, then that might suggest that a certain selection bias that could be indicated in the limitations.	We added additional details: More than 8257 pregnant women were approached to participate in this study, and 5260 were either not eligible (n=4401) or declined to participate (n=859). When a reason was provided, the majority just said that they had no interest in being in a study (n=732). Other reasons provided were personal (n=9), partner/family advice (n=38), and transportation costs associated with eight contacts (n=2).	Sample and Randomization, Lines 303-307
Line 220: 1) Was there iterative adaptation of the study interventions or ACASI over the 3 year period? 2) Did 'standard of care' or usual ANC change at all during this period?<br><br>Did the facilities host other ANC quality improvement activities, such as ANC ultrasound studies/demonstration projects, during this same period, to your knowledge?	We added these details: Throughout the study, no changes were made to the Group ANC intervention model or surveys. The only change in standard of care was the slow rollout of the transition from four recommended visits to eight contacts. However, this affected our study indirectly because we anticipated this change and adjusted the control arm so both groups had the opportunity for the same number of visits that they could attend. Also, basic obstetric ultrasound scanning services were introduced over time at the clinic; however, individuals in both arms would have benefited equally from this quality improvement activity.	Page 9, Implementation strategies section
This is not a costing or cost-effectiveness study but could the authors broadly describe the costs/inputs required for group ANC? Is it	We will discuss this more in our forthcoming implementation success paper, but we added this sentence to address this comment: Fourth, we minimized the need for additional resources and	See Implementation Strategies section, Lines 239-248

only the training of facilitators and co-facilitators? Update of ANC registers to reflect group ANC?	materials. To support future sustainability, the costs of training were absorbed by the project. Malawi now has a large cadre of trained midwives who can offer in-country training for Group ANC	
Congratulations on a well written paper concerning an important issue, I enjoyed reading it. However, I do have some concerns. Table 6 concerns important clinical outcomes and yet there is no demonstration of statistical significance. Both stillbirth and infant death seem to be considerably higher in the group ANC cohort and the only maternal death occurred in this group. Above all, women who embark on pregnancy want their babies to be born alive and survive, and of course to survive intact themselves and yet you have not considered if this difference is of statistical significance nor discussed it. I feel that this must be addressed before the paper should be published	Thank you for appreciating our first submission.<br><br>In studies involving relatively rare events, results can sometimes appear skewed, suggesting one group has experienced more or fewer incidents, despite the lack of statistical significance. Additionally, in Group ANC, the increased trust participants may have in the healthcare system could lead to more complete reporting of events, including adverse ones. We conducted statistical tests for all planned outcomes, including those in the original Table 6. Due to the rarity of these events and their non-significant *p*-values, we initially did not present them. However, based on your suggestion, these *p*-values have been added to Table 4. Except for the single maternal death, the *p*-values range from 0.14 (for miscarriages, with 17 in the Individual Care group and 10 in Group Care) to 0.74, indicating no significant differences by type of ANC.	See the Clinical Outcome portion of Table 4, which was updated to address the concerns
**Reviewer 2**		
I would also like to see some consideration as to if and how women in the group cohort were afforded the opportunity to have confidential discussions about matters of concern that they did not wish other women to know about.	We added this statement to the intervention arm: As part of the one-on-one health assessment with the midwife, women could have a confidential discussion with the midwife to address matters of personal concern. Women could have a confidential discussion with the midwife during one-on-one health assessments to address personal concerns. Health issues requiring treatment or referral were managed outside the group, and emergencies like hypertension or malaria would be addressed immediately.	See Study Interventions, Pages 8-9
Also more description as to how women were examined in a manner that preserved their privacy and dignity considering you state that most examinations occurred in the group context.	In the Study Interventions section, we describe training and practical steps that were included in addressing privacy and dignity. We wrote: As part of the one-on-one health assessment with the midwife, women could have confidential discussions to address matters of personal concern. Health issues requiring treatment or referral were managed outside the group, and emergencies like hypertension or malaria would be addressed immediately. In the Implementation Strategies, we added the following, along with citations: The second strategy was a robust training and mentoring program that emphasized fidelity and strategies to ensure respectful care [88]. Initial concerns about discussing sensitive topics or health assessments in group space often diminish as trust builds and facilitators become more experienced [11,38,92–94]. Our training drew on successful training that is sensitive and accommodates individual needs while supporting fidelity to the core components[11,12,88,95,96].	See Study Interventions section, Lines 210-214 the Implementation Strategies section, Lines 225-232
Although you have clearly described the many advantages that you found for the group cohort I would like to have read a more nuanced consideration of possible disadvantages, which may have included challenges to patient confidentiality.	In addition to addressing confidentiality above, we added sentences into the Discussion, Limitations, and Conclusion sections to not only highlight positive outcomes but also balance this by acknowledging the challenges and limitations of group care.	See Discussion, Limitations, and Conclusion sections
